# Silk Fibroin Hydrogels Could Be Therapeutic Biomaterials for Neurological Diseases

**DOI:** 10.1155/2022/2076680

**Published:** 2022-05-02

**Authors:** Chun Yang, Sunao Li, Xinqi Huang, Xueshi Chen, Haiyan Shan, Xiping Chen, Luyang Tao, Mingyang Zhang

**Affiliations:** ^1^Institute of Forensic Sciences, School of Basic Medicine and Biological Sciences, Soochow University, Suzhou, China; ^2^Department of Obstetrics and Gynecology, The Affiliated Suzhou Hospital of Nanjing Medical University, Suzhou, China

## Abstract

Silk fibroin, a natural macromolecular protein without physiological activity, has been widely used in different fields, such as the regeneration of bones, cartilage, nerves, and other tissues. Due to irrevocable neuronal injury, the treatment and prognosis of neurological diseases need to be investigated. Despite attempts to propel neuroprotective therapeutic approaches, numerous attempts to translate effective therapies for brain disease have been largely unsuccessful. As a good candidate for biomedical applications, hydrogels based on silk fibroin effectively amplify their advantages. The ability of nerve tissue regeneration, inflammation regulation, the slow release of drugs, antioxidative stress, regulation of cell death, and hemostasis could lead to a new approach to treating neurological disorders. In this review, we introduced the preparation of SF hydrogels and then delineated the probable mechanism of silk fibroin in the treatment of neurological diseases. Finally, we showed the application of silk fibroin in neurological diseases.

## 1. Introduction

Silks, popularly known for their flexibility and degradability, have been used as suture materials, and silk fibroin (SF) is a natural nonphysiological macromolecular protein extracted from boiling silk cocoons in an alkaline solution. SF is made up of a light chain (25 kDa) and a heavy chain (391 kDa). The heavy chain is the dominant component of 5263 amino acid residues, most of which are glycine (G, 45.9%), alanine (A, 30.3%), and serine (S, 12.1%). Because hydrophobic regions of short side chain acids dominate in the primary sequence, silk usually consists of *β*-sheet structures, which allow for tight packing of stacked slices of hydrogen-bonded antiparallel chains of the protein [1]. Based on these structures, SF has impressive mechanical properties to assist the development of functional tissues, especially SF from silkworms and orb-weaving spiders. Excellent biocompatibility is the premise of its application as a biomaterial. Most biomaterials should be degraded at a rate that matches the formation of the new tissue to facilitate the deposition of extracellular matrix and tissue regeneration. In addition, silk fibroin can be modified with amino acid side chain chemistry to alter surface properties and impact cell proliferation. It is also noteworthy that SF has versatility options for sterilization compared with other fibrous proteins [2]. Under high-temperature environmental conditions, it can maintain its original form and structure [3]. In summary, SF is a representative structural protein due to its features that suit a wide range of biomedical applications.

Hydrogels are a sort of polymer material with a three-dimensional (3D) network structure, which is formed by chemical or physical cross-linking of hydrophilic polymer chains in an aqueous solution [4]. It absorbs large amounts of water molecules and swells but does not dissolve. Owing to their excellent water solubility, biocompatibility, ductility, and deformability, hydrogels are advanced material platforms in biomedical therapies [5]. However, the single cross-linking method and the lack of an effective energy dissipation mechanism make the mechanical properties of traditional hydrogels poor, which limits their large-scale applications. The structure and conformation of SF strongly affect its mechanical properties, and the formation of *β*-sheets in the structure of SF enhances its mechanical properties [6, 7]. Based on the adjustability of SF structure by physical or chemical treatment and cross-linking strategies of SF hydrogels with other biological materials, not only did SF hydrogels solve this problem but also the application value of SF hydrogels in tissue regeneration and treatment of tumors was found.

Neurological diseases are caused by a variety of factors that lead to organic or functional disorders of the brain and spinal cord. Because of the irreversibility of neurons, the treatment and prognosis of related diseases are not satisfactory. Despite attempts to propel neuroprotective therapeutic approaches, effective advances in neuroregeneration have still not been reached. It has been suggested that silk fibroin can promote neuroregeneration, and the development of silk fibroin-based biomaterials indicates a new frontier to apply in neuroregenerative therapies. This review is aimed at introducing advances in SF hydrogels. Pathophysiological mechanisms and applications of SF in neurological disorders will also be examined.

## 2. Preparation of SF Hydrogels

Silk fibroin can be processed in versatile patterns, specifically aqueous-based platforms, and hydrogels are examples. When subjected to controllable physical factors such as temperature, solution concentration, shear force, and ions, SF can spontaneously aggregate or self-assemble into hydrogels [8]. Chemical cross-linking can also accelerate the gel by adding functional groups to form covalent bonds. There are sundry methods to fabricate SF hydrogels, and these techniques are important factors affecting their properties.

### 2.1. Physical Methods

#### 2.1.1. Temperature

It has been shown that temperature plays a critical role in influencing protein aggregation, which depends to a large extent on the hydration of the system [9]. Increasing temperature can increase the average kinetic energy of the particles in the system and then increase the opportunity for effective collision, which is beneficial to the assembly of macromolecules. On the one hand, the dense accumulation of water around the protein encourages an increase in dehydration in the hydrophobic region and enhances hydrophobic interactions and cross-linking. On the other hand, it can perturb the free energy state of macromolecules and promote protein unfolding and hydrophobic region exposure [10]. These events work together to assemble and aggregate proteins.

#### 2.1.2. Shear Forces

Shear force is applied to silk protein solution to cause fluid rotation and stretching, which changes the macromolecule orientation and polymer chain stretching [11]. It impacted the end-to-end distance and arrangement of the polymer chain, propelling the fluctuation of concentration and the intermolecular force [12]. The extended vortex-induced method is an easy way to make SF hydrogels with appropriate mechanical properties [13]. With increasing vortex time, the molecular conformation and intermolecular self-assembly changed with increasing protein *β*-sheet content, which may reflect the viscoelasticity of SF hydrogels.

#### 2.1.3. Ultrasound

Ultrasound can affect other physical stimuli, such as local temperature raising, shear force extension, and changes in gas-liquid interface equilibrium, to promote SF rapid gelation [14, 15]. Von et al. reported that ultrasonication is a more effective way to produce hydrogel composite systems that are stable, homogenous, and well blended without phase separation [16]. Noticeably, although the gel time is controllable and it did not introduce other immunogenic substances, it requires high preparation conditions and has poor reproducibility.

#### 2.1.4. Electric Field

Several studies are prospective on how to apply an electric field to prepare electroactive SF hydrogels. The extra electric field can indirectly affect the local pH by increasing the proton concentration in the positive electrode, thereby regulating the aggregation of silk proteins [13]. Leisk et al. [17] discovered that in 8.4% SF solution, 25 V direct current was inserted into the gel on the positive electrode of platinum, and the gel had excellent adhesion. This study also demonstrates that the self-assembly of SF hydrogels is reversible. When the electrode is exchanged, the SF hydrogel reaggregates on the new positive electrode.

### 2.2. Chemical Methods

#### 2.2.1. Organic Solvent Induction

At present, classical precipitators mainly include salt, organic solvents, and surfactants. Adding salt to SF aqueous solution can increase the ionic strength of the solvent and enhance the ability to combine with water, thus changing the interaction between the protein molecules to promote the internal binding of the protein [18]. Numerous studies have confirmed that the formation of SF hydrogels can be regulated by organic solvents. First, the addition of an organic solvent can alter the dielectric properties of the solvent and reduce the solubility of water, thus promoting supersaturation of the solution [18, 19]. Simultaneously, organic solvents generally with strong polarity destroy the intermolecular hydrogen bonds and electrostatic bonding, increasing molecular chain unfolding and *β*-sheet generation, and then the chain segments rearrange and self-assemble [20]. Among those organic solvents, alcohol is the most common [21, 22]. Besides, glycerol [23] and ethylene glycol diglycidyl ether (EGDE) [24] are can be used as well. Surface agents refer to making the interface state of the solution change dramatically, such as poloxamer 407 [25] and sodium dodecyl sulfate (SDS) [26].

#### 2.2.2. pH

pH is one of the crucial parameters to readjust SF hydrogels. It can convert the ionization state of the amino acid residues on the gel surface and can promote gel formation when the pH is on the brink of the isoelectric point (pI). In a study, it was impressive to note that adjusting the pH allows the solution to transform between gel and sol, but only under transient exposure conditions [27]. Mechanistically lowering the pH inhibits the ionization of the acidic amino acid so that the balance between repulsion and attraction is crippled and the hydrophobic interaction is strengthened, resulting in a weaker bond [27]. Nevertheless, under acidic conditions for long periods, the denaturation of SF and extended intramolecular and intermolecular hydrogen bonds promote the formation of large amounts of *β*-sheets, forming stable gels [27, 28].

#### 2.2.3. Carbon Dioxide

Carbon dioxide (CO_2_), as a volatile acid, can be a novel way to prepare SF hydrogels without surfactants or chemical cross-linking agents. Floren et al. [29] obtained stable hydrogels under high-pressure CO_2_ treatment for less than 2 hours with tremendous physical properties such as porosity, sample homogeneity, swelling behavior, and compressive features. However, whether high pressure has a promoting effect on gel formation is still complex and worth exploring. Another study experimented with low-pressure CO_2_, in which gel time was briefly within ten minutes because of very small gas bubble-to-liquid path lengths, significantly shorter than the high-pressure condition (120 minutes) [30]. Therefore, high pressure does not seem to necessarily reduce the gel time.

#### 2.2.4. Photocuring

Photocuring is a method of preparing hydrogels by chemical cross-linking. The presence of light energy, including ultraviolet (200-400 nm) and visible light (400-800 nm), provides photons to photoinitiators, and then they absorb the energy of the photons to split into free radical molecules. These molecules react with the vinyl bonds in the prepolymer, resulting in chemical cross-linking of the polymer chain, namely, free radical-initiated chain polymerization [31, 32]. Photooxidation is advantageous in maintaining the protein structure and eliminating the need for chemical modification. In contrast, methacrylate is better in terms of photo-cross-linking efficiency by organic solvents, such as acetone and ethanol [33].

#### 2.2.5. Chemical Cross-Linking and Modification

**Figure 1:**
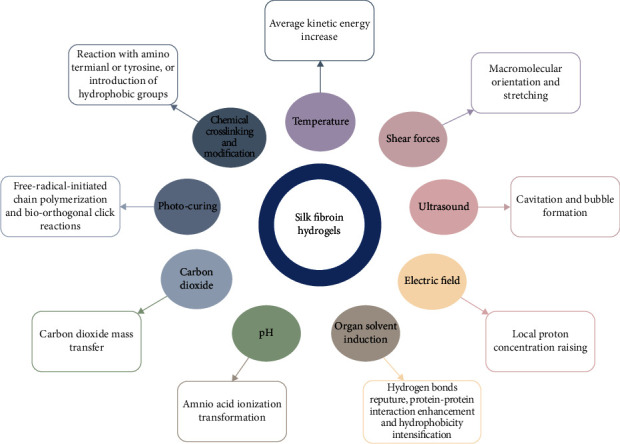
The preparation of SF hydrogels.

Chemical cross-linking agents, including glutaraldehyde, genipin, diepoxide, and horseradish peroxidase (HRP) enzyme plus hydrogen peroxide (H_2_O_2_), have been used in practice [8, 34–36]. Chemical cross-linking and modification are fabricated to prepare hydrogels by forming covalent bonds according to the molecular structure of SF. Most of them are located in the amino group of SF. Chemical modification can involve the chemical properties of SF to achieve SF hydrogel functionalization. Modifications such as sulfation of tyrosine, azo-modified tyrosine, and arginine masking provide sites for growth factors, cell-binding domains, and other polymers to attach to SF, expanding the range of biomaterial applications [37]. Herein, regulatory factors of SF hydrogels are diverse, which interact with each other (Figure 1).

Silk fibroin hydrogels can be processed in versatile patterns. The methods to fabricate SF hydrogels, including physical and chemical methods, are important factors affecting the properties of SF. SF hydrogels exhibited networks morphology with *β*-sheet structure through the mutual influence of each regulatory factor.

### 2.3. Blend Hydrogels

Poor elasticity, low water retention ability, and lack of a cell attachment sequence limit the usage of SF. As long as the materials have no vital negative biological impact, both natural and synthetic polymers can be utilized to fabricate hydrogels, which result in rapid gelation and high biological activity. Natural polymers have higher biocompatibility, excellent biodegradability, and no toxicity. So far, collagen, chitosan, gelatin, silk fibroin, alginate, cellulose, hyaluronic acid (HA), and starch, alone or in combination, have been widely used in tissue engineering. Polymerization with gelatin can improve biological activity, such as cell attachment, diffusion, and proliferation [38]. In recent years, tyramine modification catalysis by enzymes has gradually become the emerging trend of HA involvement in SF hydrogels. HA content can affect the extracellular matrix and upregulate matrix protein expression, and HA-Tyr-SF is suitable for tissue engineering and drug delivery as an injectable biomaterial [39]. Apart from the abovementioned polymers, the combination of SF with cellulose, alginate, and chitosan has exceptional ability in tissue regeneration engineering, drug delivery, wound healing, and other fields [40–42]. SF can be mixed also with synthetic polymers. Silk fibroin-based mixtures with poly (ethylene glycol), poly (vinyl alcohol), polyacrylamide, polycaprolactone, poly (lactic-co-glycolic acid), polyurethane, and polylactide have been reported [43]. Adjusting the properties of macromolecular polymers, such as molecular weight transitions or chemical modifications, directly urges the mechanical, degradability, and physical capabilities of synthetic polymers, all of which afford functionality and task-specific biomaterial traits in tissue regeneration and wound healing [44]. Designing SF hydrogels with controllable mechanical properties, degradation rate, and biocompatibility per regenerated tissue is key to regenerative therapies to better achieve individualized therapeutic goals.

## 3. Pathophysiological Mechanisms of SF Hydrogels

### 3.1. Neuroregeneration

**Figure 2:**
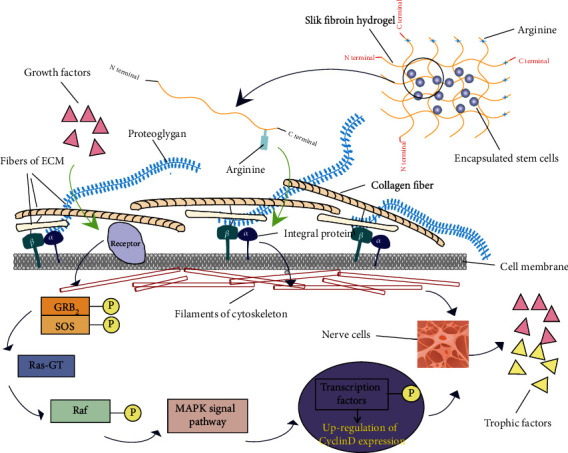
SF hydrogels play a 3D microenvironment akin to natural ECM.

Regeneration and repair of nerve tissue after brain injury are difficulty in the treatment and improvement of neurological diseases. Regenerative therapies based on active biomaterials and encapsulated therapeutic stem cells have profound therapeutic potential. As a naturally energetic material, SF has nerve biocompatibility and can support cell adhesion, proliferation, and neural differentiation. Hydrogels can modulate the mechanical stiffness of brain tissue and replace the damaging environment, acting as cell carriers or growth factor delivery vehicles. Seed cells, such as embryonic stem cells, neural stem cells, and mesenchymal stem cells, completed cell adhesion, cell proliferation, and cell differentiation in the ECM microenvironment mimicked by SF hydrogels and achieved tissue regeneration under the interaction of growth factors and nutrients [45–47]. The biochemical factors fixed on the ECM regulate local concentrations and influence cell proliferation and differentiation. Although SF hydrogel scaffolds can mimic the 3D microenvironment of natural ECM to facilitate nutrient exchange and provide mechanical protection, the effects of the secretive activity of encapsulated stem cells remain to be seen. Martín-Martín et al. demonstrated that MSCs encapsulated in SF hydrogels may induce the secretion of several neurogenic and angiogenic factors more than the nonencapsulated group, such as brain-derived neurotrophic factor (BDNF), vascular endothelial growth factor (VEGF), and SDF-1 protein [48]. Biomaterial surfaces can present nanoscale topographical cues which influence neuronal differentiation and process outgrowth. Bai et al. designed SF nanofiber hydrogels and complex three-dimensional (3D) porous structures to mimic the elastic modulus and topography by altering the annealing process [46]. They demonstrated that 50% methanol promoted the differentiation of NSCs into astrocytes, while 80% methanol inhibited this phenomenon, and the number of caspase 3^+^ cells in the treatment groups was decreased, suggesting that the topography and mechanical evaluation brought by annealing treatments prevented or delayed NSC apoptosis [46]. In this context, we briefly summarize that SF hydrogels may play a 3D microenvironment akin to natural ECM, facilitating mechanical protection and nutrient exchange (Figure 2).

Recognition between the arginine sequence in SF and integrin on the cell membrane mediates cell adhesion to the ECM and then affects cytoskeletal motion. On the other hand, growth factors, such as PDGF and FGF, combined with receptors, induce cell proliferation through the MAPK signaling pathway. In parallel, they also induce the differentiation of encapsulated stem cells into the neuronal cells, which then secrete related growth factors and nutritional factors to accelerate nerve tissue regeneration and improve tissue repair.

### 3.2. Regulating Inflammation

Active inflammatory activity after brain injury causes irreversible damage to the brain, which is not conducive to the treatment and postoperative recovery of patients. SF hydrogels play an anti-inflammatory role because they are regarded as mechanical barriers to block the negative regulation of inflammatory factors (such as TNF-*α*) interacting with anti-inflammatory factors and neurotrophic factors. More importantly, it upregulates the expression of the anti-inflammatory factor TGF-*β*, which in turn allows microglia to differentiate into an anti-inflammatory phenotype and repair brain damage [48]. This may be linked to cytoskeletal abnormalities, oxidative stress responses, and hypoxic microenvironments [48]. Some researchers have combined anti-inflammatory active substances or drugs with SF hydrogels to exert anti-inflammatory effects. After introducing biliverdin, a precursor of bilirubin, into the SF hydrogel systems for incubation, the expression of the inflammatory factors TNF-*α* and interleukin-1 (IL-1) was significantly downregulated at 7 and 14 days [49]. In another study, betamethasone was loaded into tyramine-modified gellan gum with silk fibroin (Ty-GG/SF), hydrogel and the concentration of TNF-*α* was undoubtedly decreased [50]. In contrast to traditional drug administration, this method can enhance the local concentration of drugs in the inflammatory environment to exert its efficacy, preventing normal tissues from the toxic effect of drugs on cells and tissues.

### 3.3. Sustained Drug Release

Owing to its unique material properties, stabilization effects, and tight controllability, silk fibroin hydrogel represents a promising, controlled, sustained drug release from fully degrading implants. Betamethasone has a short half-life, meaning that it needs to inject with repeated doses to maintain the active drug concentration. Oliveira et al. designed composite Ty-GG/SF hydrogels that have more *β*-sheet structures, better mechanical properties, and more resistance to enzymatic degradation than pure SF hydrogels [50]. Thus, it can improve the pharmacokinetic characteristics of the controlled release of betamethasone over time, prolong the duration of treatment, reduce the frequency of administration, and decrease the toxicity of drugs. As a drug-loading platform, SF hydrogels have the problem of initial drug release. However, this does not necessarily lead to bad results. Some scholars have concluded that the addition of SF can enable the rapid release of hydrophobic drugs, providing a new technical means for the rapid increase in local drug concentration in some diseases [51]. Other studies have shown that adjusting SF and other drugs/polymers ratios can lead to sustained-release capabilities of risperidone. At ratios of 1 : 3 and 1 : 6, SF/methanol hydrogels contained risperidone, which could be made public for 25 days, but the former had better structural stability [52]. In conclusion, SF-based platforms that permit both sustained and local release of factors represent an attractive approach for efficient delivery of the factor to the brain. Therefore, emphasis should be placed on the combination of material characteristics, drug effects, clinical therapeutic purposes, and expected efficacy, and appropriate SF hydrogels should be selected as drug-loading vehicles.

### 3.4. Antioxidative Stress

Oxidative-antioxidant dynamic balance in the central nervous system (CNS) affects normal brain function. Excessive production of endogenous or exogenous oxides and/or inadequate function of the antioxidant response system will lead to the accumulation of adverse reactive oxygen species (ROS), leading to oxidative stress response and making brain tissue overly susceptible to damage [53]. Oxidative stress is the main cause of neurodegenerative diseases due to the oxidation of DNA, lipids, and proteins, thereby inducing a secondary cellular response. Antioxidants can be used to scavenge free radicals, and SF biomaterials can be regarded as good carriers of antioxidants. In a study, SF nanoparticles (SFSNPs), which were infiltrated into the antioxidant sulforaphane, effectively reduced the increase of ROS levels caused by hydrogen peroxide- (H_2_O_2_-) mediated oxidative stress [54]. Based on the excellent mechanical properties and biocompatibility of SF hydrogels, we speculated that SF hydrogels could form a physical barrier to protect antioxidants from removal, prolong the preservation time, and exert a lasting antioxidant stress effect. On the other hand, SF hydrogels equipped with stem cells or growth factors and other active substances can be developed to provide a living space for antioxidant enzymes and simulate the physiological antioxidant system.

### 3.5. Regulating Cell Death

Cell death is important in both physiological and pathological conditions. Cell death in the brain contains the following characteristics: (1) neurons are permanent cells with limited regenerative capacity; (2) because of the various types of ion channels in nerve cells, damage to them may result in various death patterns; and (3) the high consumption of ATP in brain tissue and the modest turnover of ATP may induce the death of ischemic hypoxia stimulation [55]. Currently, cell death can be divided into programmed and nonprogrammed cell death. In general, except necrosis, apoptosis, autophagic cell death, ferroptosis, and pyroptosis constitute programmed cell death. The control and regulation of the apoptotic events that occur through SF have been reported recently. Anticancer activity of SF peptides (SFPs) has been proven to be associated with the inhibition of tumor cell proliferation, induction of apoptosis, and cell cycle arrest [56]. NSCs encapsulated by SF nanofiber hydrogels exhibited a reduced number of caspase 3^+^ cells, suggesting that SF prevented or delayed NSCs from undergoing apoptosis [46]. Our team is currently investigating other cell death pathways. Overall, we are positive about the SF prospect of regulating cell death, including pyroptosis and ferroptosis.

### 3.6. Hemostasis

Bleeding is a common clinical symptom caused by acute trauma. The amount of blood loss, timing, and timely intervention are closely related to the patient's prognosis. Effective hemostasis and hemostatic materials play an important role in controlling blood loss. Current medical hemostatic materials have side effects, such as wound infection, inflammatory injury, and body allergies caused by gelatin [57]. Studies have begun to consider SF an alternative material with good biocompatibility and hemostatic properties. Visceral injury is very common in trauma and is easily accompanied by blood loss. A team constructed an animal model of liver injury (wound size: 1.5 cm × 1.0 cm × 0.2 cm) to evaluate the hemostasis of SF by hemostasis time and blood loss. The results showed that the addition of SF, as part of the material composition, improved hemostatic performance and was superior to the gelatin group and the blank group [58]. SF can rapidly gel to block the bleeding site as a physical barrier and induce platelet adhesion and aggregation and enhance platelet and fibrinogen interactions while maintaining platelet activity [58]. This performance is similar to that of the endogenous platelet agonist ADP, which induces blood coagulation and has a hemostatic effect. Research on the hemostatic performance of SF is just beginning, and the specific mechanism of SF on physiological hemostasis, such as the mechanism affecting the release of coagulation factors, still needs more research explanation. The role of silk fibroin hydrogels in brain hemostasis can be studied in the future.

## 4. Potential Roles of SF Hydrogels in Neurological Diseases

Laboratory applications of SF cover a wide range of fields, with outstanding performance in vivo and/or in vitro experiments (Table 1). Clinical trials have been carried out in some areas dabbling in neurological diseases, but the results are less satisfactory than had been anticipated (Table 2). The potential value of SF hydrogels in neurological diseases is systematically described in Table 1.

### 4.1. Cerebral Stroke

Acute cerebral strokes, also known as cerebrovascular accidents, are broadly classified as either ischemic or hemorrhagic. The time window of treatment is narrow after the onset of stroke, accompanied by severe complications such as angioedema, intracranial hemorrhage, or systemic massive hemorrhage, which is a leading cause of serious long-term disability [73]. Therefore, early identification and intervention affect the clinical evolution of stroke. The safety of silk fibroin in the brain has been investigated. Fernandez-Garcia et al. reported that the striatal injection of SF hydrogel was reasonably well tolerated by the animals because the survival rate exceeded 90% and was similar to that of the saline group [74]. The authors also demonstrated that SF hydrogel encapsulated stem cells reduced the damaged cortical infected area and exerted progressive neuroprotective effects in ischemic stroke [75]. Another study explained the excellent spatial consistency of SF hydrogels in filling the stroke cavity and provided a reliable matrix for regeneration without significant inflammation mediated by microglia/macrophage activation [76]. Lim et al. developed an injectable gelatin hydrogel containing epidermal growth factor (Gtn-ECF), which can effectively repair the defective tissue and restore nerve function after intracerebral hemorrhage (ICH) [77]. However, the application of SF hydrogel in hemorrhagic stroke is still in the theoretical stage, and the role of SF hydrogel in ICH needs to be investigated.

### 4.2. Traumatic Brain Injury

Traumatic brain injury (TBI), also known as an intracranial injury, causes functional impairment of key brain areas under the action of strong external forces, which has brought a huge burden to families and society and has become a major problem endangering public health security [78]. After TBI, the microenvironment of ischemia and hypoxia caused by glial hyperplasia, inflammation, and lack of neurotrophic factors inhibits the regeneration and repair of brain tissue. The development of SF hydrogel either alone or in combination with other therapies will be a reasonable and innovative choice in the field of TBI treatment. Moisenovich et al. reported that transplantation of silk fibroin microparticles into the injury locus of the brain resulted in a decrease in damage volume, as well as the restoration of sensorimotor functions [79]. Tang-Schomer et al. showed that silk fibroin films can be utilized to evaluate drug actions in both in vitro and in vivo studies of brain injury with greater efficiency than existing approaches [80]. Silk film-delivered necrostatin reduced cell necrosis in the TBI model, which is consistent with intracerebroventricular delivery of necrostatin in reducing histopathology and improving functional outcomes [81]. A combination of SF/collagen/human umbilical cord mesenchymal cells (hUMSCs) coculture (CB group) has been implanted into TBI canine models, showing great potential for treating TBI. Compared with the stem cells group (SC group) and the collagen/SF group (CS group), the CB group displayed a significant anti-inflammation effect and repair of cerebral cortical motility after TBI [82]. Although the study confirmed the potential of SF hydrogels and stem cells regeneration therapies for clinical applications, questions remain to be considered. For example, the regulation of stem cells survival and targeted differentiation, the specific mechanism of injury repair, and the optimal time window for implantation necessitated further exploration and discussion [83].

### 4.3. Brain Tumors

Glioblastoma (GBM) is the most frequent tumor in the central nervous system and has highly diffuse malignant infiltration behavior. Chemotherapy, a routine operation for the clinical treatment of GBM, lacks the specificity of systemic administration, and side effects that occur during chemotherapy treatment seriously affect the homeostasis of the body in patients, which are not conducive to treatment and recovery [84]. Moreover, hydrophobic chemotherapeutic drugs account for a large proportion of chemotherapeutic drugs, and their solubility in water medium is scant. SF contains more hydrophobic areas, and cross-linking between SF and hydrogels can enhance hydrophobicity to directly transport hydrophobic drugs to the tumor site. Xu et al. constructed indocyanine green-SF nanoparticles (ICG-SFNPs) for photothermal therapy of glioblastoma, which can form local high temperatures and cause a large area of tumor necrosis [85]. Recently, Ribeiro et al. reported that the crystalline SF hydrogels converted into *β*-sheet structure induced the formation of TUNEL-positive apoptosis in a human neuronal glioblastoma cell line (U251) [86]. Wang et al. showed that a silk fibroin microneedle (SMN) patch loaded with chemotherapeutic agents (thrombin and temozolomide) and targeted drug (bevacizumab) induces rapid drug delivery and results in decreased tumor volume and increased survival rate in GBM mice [87]. From the foregoing description, we boldly assume that SF hydrogels can act as a physical barrier to resist the degradation of encapsulated drugs. In parallel, enhancement of hydrophobicity is beneficial for SF hydrogels as drug delivery systems for the targeted therapy of GBM.

### 4.4. Neurodegenerative Diseases

Neurodegenerative diseases are characterized by progressive loss of vulnerable populations of neurons and histopathological findings of abnormal conformational change of self-proteins, including amyloidosis, tau protein, alpha-synuclein, and transactivation response DNA binding protein 43 (TDP-43) [88]. The highly repetitive GAGAGS in SF can be analogous in structure to VGGVV in amyloidosis *β*42 peptide and VGGAVVAGV in alpha-synuclein [89]. Simultaneously, the silk I structure (random coil and helix-like forms) turns into silk II (beta-sheet and beta-sheet-like forms), a nucleation-dependent conformation transition principle, that bears extreme resemblance to that of neurodegenerative-related proteins [90]. The antioxidant, neuroprotective, and acetylcholinesterase inhibitory mechanisms of silk proteins could prove promising in the treatment of neurodegenerative diseases [91]. However, the application of SF hydrogel in neurodegenerative diseases is still in the theoretical stage and needs to be investigated.

### 4.5. Traumatic Spinal Cord Injury

Traumatic spinal cord injury (TSCI) is a devastating central nervous system disease and can be divided into primary injury (initial mechanical injury) and secondary injury, including ischemia, oxidative stress, axonal degeneration, and cell death triggered by inflammatory processes [92]. The involvement of SF in post-SCI has been demonstrated in several studies [71, 93, 94]. In vitro culture of human amniotic epithelial cells (AECs) implanted with SF scaffolds showed active proliferation and migration, which improved motor function after SCI [95]. Bone mesenchymal stem cells (BMSCs) implanted in SF scaffolds confirmed the excellent performance in axonal regeneration, myelination, and functional recovery in SCI rat models [96]. Drug-loaded injectable SF scaffolds for the treatment of SCI have also great potential for spinal cord regeneration. Han et al. reported that metformin-loaded silk fibroin microsphere could improve the growth and spreading behavior of cortical neurons after SCI [97]. Taken together, the application of SF hydrogel in TSCI is promising and worthy of further exploration.

## 5. Conclusion and Limitations

In summary, with in-depth research, the silk fibroin hydrogels have transformed from simple and independent structures to functionalized cross-linked forms with other polymers by physical or chemical methods. It is widely accepted that SF hydrogels loaded with cells and growth factors have great potential to address the challenge of regenerating neuronal cells. The prospects of using silk fibroin hydrogels alone and their blends are quite exciting in neurological disease due to the positive results achieved in vitro and in vivo. Despite the promising results mentioned earlier, there are still some important issues to be addressed in the future. First, the major disadvantages of silk fibroin hydrogels are their poor mechanical properties and swelling behavior, which are very important parameters in biomedical applications. To improve the properties of SF hydrogels, silk fibroin has been blended with various other polymers. Therefore, the potential long-term toxicity and the nonbiodegradability of blended SF hydrogel should be further investigated. Second, the specific effects of SF hydrogel on cells, tissues, or organs and their metabolic pathway in vivo remain unclear and require further studies. Third, the cellular/molecular mechanisms behind the neuroprotective ability of SF hydrogel should be further investigated. It is expected that the silk fibroin hydrogels loaded with or without seed cells or drug agents may be the most effective treatment for brain disease and are subject to future clinical trials. However, at present, the functionalization of the silk fibroin hydrogels in the neurological disease is still in its infancy; thus, many aspects are still needed to be developed and improved. In summary, silk fibroin hydrogels have great prospects of expanding their niche in the field of brain tissue regeneration, repairing damaged brain tissue, and improving neurologic recovery after injury.

## Figures and Tables

**Table 1:** List of the applications of SF or SF blends.

Application	Materials	In vitro	In vivo	Cite
Cartilage tissue engineering	GMA/SF	Human chondrocytes	Female athymic mice, rabbits	[59]
Bone defect repair	n-HA-GO/SF	Rabbit bone marrow mesenchymal stem cells (BMSCs)	Male mice critical-sized bone defect models	[60]
Ligament reconstruction	SF/HA-SF	Rat BMSCs	Rabbit models of ACLR	[61]
Wound healing	CF@GO/SF	Human fibroblast (L929) cells	Rat models of wound	[62]
Reconstitution of cardiac function	SF	Rat quiescent ventricular cardiomyocytes	Left ventricles of rats	[63]
Vascular remolding	SF/GT	Mouse BMSCs	/	[64]
Trachea reconstruction	SF/CVM	Human-induced pluripotent stem cells (hiPSCs)	Porcine tracheal defect models	[65]
Artificial cornea engineering	PVA/SF/n-HA/GP	Human corneal fibroblasts (HCFs)	/	[66]
Enamel regeneration	SF/HA	/	/	[67]
Hepatic tissue engineering	SF	Stem cells from human exfoliated deciduous teeth (SHED)	/	[68]
Bladder reconstruction	SF	Adipose-derived stem cells (ASCs)	Rat bladder augmentation models	[69]
Nucleus pulposus replacement	SF/PU	/	Rabbits	[70]
Nerve regeneration	Collagen/SF	Human umbilical cord mesenchymal stem cells (hUC-MSCs)	Rat models of SCI	[71]
Tumor therapy	Biliverdin/SF	/	Mice tumor models	[49]
Drug delivery	DEX/CSNPs/SF	L929 fibroblast cell line	/	[72]

GMA: glycidyl methacrylate; n-HA: nanohydroxyapatite; GO: graphene oxide; ACLR: anterior cruciate ligament reconstruction; CF: ciprofloxacin; GT: gelatin-tyramine; CVM: collagen vitrigel membrane; GP: genipin; PU: polyurethane; SCI: spinal cord injury; DEX: dexamethasone; CSNPs: chitosan nanoparticles.

**Table 2:** List of the published clinical trials that highlight the application of silk-based materials.

No.	Intervention/treatment	Condition/disease	Number of participants	Compete date	Clinical trial identifier
1	Device: The SF patch or paper patch	Diseases of the ear and mastoid process	60	June 2012	KCT0000305
2	Device: HQ® matrix medical wound dressing Device: Sidaiyi® wound dressing	Donor site wound	71	September 2014	NCT01993030
3	Device: silk fibroin with bioactive coating layer dressing	Late complication from skin graft; infection of skin donor site; impaired wound healing; pain, intractable	29	May 2015	NCT02091076
4	Bilayer scaffold composed of amniotic membrane and silk fibroin	Diabetes mellitus; Wagner ulcer grade II	20	February 2018	IRCT2016071328903N1
5	Absorbable SF membrane	Alveolar ridge preservation after tooth extraction	65	December 2018	ChiCTR1800016759
6	Autologous chondrocytes seeded in SF scaffolds	Osteochondral defects	15	July 2019	IRCT2017062434731N1
7	SF membrane combined with xenograft	K053-chronic periodontitis	15	December 2019	CTRI/2018/12/016509
8	SeriossÂ® (SF scaffold)	Patients undergoing surgery for bone void filling	10	/	CTRI/2021/01/030589
9	Device: Silk Bridge	Peripheral nerve injury digital nerve hand	4	March 2021	NCT03673449
10	Silk sericin dressing with collagen	Wound heal; wound surgical; donor site complication	30	December 2021	NCT04743375
11	Procedure: Silk microparticle filler injection	Vocal cord paralysis unilateral; dysphonia; dysphagia, oropharyngeal	100	September 2024	NCT03790956

Source: data retrieved from International Clinical Trials Registry Platform.
